# Impact of regadenoson-induced myocardial creep on dynamic Rubidium-82 PET myocardial blood flow quantification

**DOI:** 10.1007/s12350-019-01649-4

**Published:** 2019-02-20

**Authors:** S. S. Koenders, J. D. van Dijk, P. L. Jager, J. P. Ottervanger, C. H. Slump, J. A. van Dalen

**Affiliations:** 1 Department of Nuclear Medicine, Isala hospital, PO Box 10400, 8000 GK Zwolle, The Netherlands; 2 Department of Medical Physics, Isala Hospital, Zwolle, The Netherlands; 3 Department of Cardiology, Isala Hospital, Zwolle, The Netherlands; 4 MIRA: Institute for Biomedical Technology and Technical Medicine, University of Twente, Enschede, The Netherlands

**Keywords:** Myocardial blood flow, PET myocardial perfusion imaging, ^82^Rb, Myocardial creep, Regadenoson

## Abstract

**Background:**

Repositioning of the heart during myocardial perfusion imaging (MPI) using Rubidium-82 (Rb-82) PET may occur when using regadenoson. Our aim was to determine the prevalence and the effect of correcting for this myocardial creep on myocardial blood flow (MBF) quantification.

**Methods:**

We retrospectively included 119 consecutive patients who underwent dynamic rest- and regadenoson-induced stress MPI using Rb-82 PET. The presence of myocardial creep was visually assessed in the dynamic stress PET series by identifying differences between the automatically drawn myocardium contour and the activity. Uncorrected and corrected stress MBFs were compared for the three vascular territories (LAD, LCX, and RCA) and for the whole myocardium.

**Results:**

Myocardial creep was observed in 52% of the patients during stress. Mean MBF values decreased after correction in the RCA from 4.0 to 2.7 mL/min/g (*P * < 0.001), in the whole myocardium from 2.7 to 2.6 mL/min/g (*P * = 0.01), and increased in the LAD from 2.5 to 2.6 mL/min/g (*P * = 0.03) and remained comparable in the LCX (*P * = 0.3).

**Conclusions:**

Myocardial creep is a frequent phenomenon when performing regadenoson-induced stress Rb-82 PET and has a significant impact on MBF values, especially in the RCA territory. As this may hamper diagnostic accuracy, myocardial creep correction seems necessary for reliable quantification.

**Electronic supplementary material:**

The online version of this article (10.1007/s12350-019-01649-4) contains supplementary material, which is available to authorized users.

## Spanish Abstract

**Antecedentes:**

El movimiento del corazón durante la imagen de perfusión miocárdica usando Rubidio-82 (Rb-82) PET puede ocurrir cuando se usa regadenosón. Nuestro enfoque fue determinar la prevalencia y efecto de la corrección de este Artefacto de movimiento en la cuantificación del flujo sanguíneo miocárdico.

**Métodos:**

Se incluyeron de forma retrospectiva 119 pacientes que se realizaron estudio dinámico en reposo y estudio de perfusión miocárdica en estrés inducido por regadenosón usando Rb-82 PET. La presencia de el artefacto de movimiento fue evaluado visualmente en el estudio en serie de PET dinámico en estrés identificando las diferencias entre el contorno miocárdico dibujado automáticamente y la actividad. La perfusión miocárdica en estrés corregida y sin corregir se compararon para los tres territorios vasculares (Descendente Anterior, Circunfleja, Coronaria Derecha) y para todo el miocardio.

**Resultados:**

El artefacto de movimiento miocárdico se observo en un 52% de los pacientes durante el estrés. El promedio de los valores de flujo sanguíneo miocárdico disminuyen después de la corrección en la arteria coronaria derecha de 4.0 a 2.7 ml/min/g (p < 0.001), en todo el miocardio de 2.7 a 2.6 mL/min/g (p = 0.01) y un aumento en la arteria descendente anterior de 2.5 a 2.6 mL/min/g (p = 0.03) y se mantuvo comparable en la arteria circunfleja (p = 0.3).

**Conclusiones:**

El artefacto de movimiento miocárdico es un fenómeno frecuente cuando se realiza la prueba de estrés inducido con regadenosón Rb-82 PET y tiene un impacto significativo en los valores de flujo sanguíneo miocárdico, principalmente en el territorio de la coronaria derecha. Esto puede disminuir la precisión diagnostica, la corección del artefacto de movimiento es necesario para una cuantificación confiable.

## Chinese Abstract

**背景:**

铷-82 (Rb-82) PET心肌灌注成像 (MPI) 过程在使用类伽腺苷时可能会发生心脏重定位。我们的目的是确定心肌蠕动 (myocardial creep) 校正对心肌血流灌注定量的影响及其普遍性。

**方法:**

我们连续回顾了119 名使用 Rb-82 PET 进行动态的静息与类伽腺苷负荷MPI的患者。通过识别自动绘制的心肌轮廓和心肌活动之间的差异, 在动态负荷 PET 检查中肉眼评估心肌蠕动的存在。分别在已校正和未校正的负荷 MBF上, 对三个血管区域 (LAD, LCX, RCA) 和整个心肌灌注情况进行比较。

**结果:**

负荷检查中, 52% 的患者观察到心肌蠕动。 RCA 校正后平均MBF值从4.0降至 2.7 mL / min / g (p <0.001), 整个心肌从 2.7降至 2.6 mL / min / g (p = 0.01) , LAD 从 2.5 升至 2.6mL / min / g (p = 0.03) 并且在LCX中保持不变 (p = 0.3) 。

**结论:**

类伽腺苷负荷 Rb-82 PET 检查中, 心肌蠕动是一种常见的现象, 并且对 MBF 值有显著影响, 特别在 RCA 区域。这可能妨碍诊断准确性, 因此心肌蠕动校正是准确定量的必要条件。

## French Abstract

**Contexte:**

Un repositionnement du cœur lors de l’imagerie de perfusion myocardique (MPI) à l’aide de la TEP au rubidium-82 (RB-82) peut survenir lors de l’utilisation du regadenoson. Notre objectif était de déterminer la prévalence et l’effet de la correction du fluage sur la quantification du débit sanguin myocardique (DSM).

**Méthodes:**

dans cette étude rétrospective, nous avons inclus 119 patients consécutifs ayant beneficié d’un examen au repos suivi d’un stress pharamacologique au  régadénoson en utilisant le RB-82. La présence de fluage myocardique a été évaluée visuellement sur les images TEP de stress dynamique en utilisant les différences entre les contours automatiques et les images myocardiques. Les DSM de stress non corrigés et corrigés ont été comparés pour les trois territoires vasculaires (coronaire gauche-CG, la circonflexe-CX, et coronaire droite-CD) et pour l’ensemble du myocarde.

**Résultats:**

le fluage myocardique a été observé chez 52% des patients sur les examens de stress. Les valeurs moyennes de DSM ont diminué après correction dans le territoire de la CD de 4,0 à 2,7 mL/min/g (p < 0,001), dans le myocarde entier de 2,7 à 2,6 mL/min/g (p = 0,01) et augmenté dans le territoire de la CG de 2,5 à 2,6 mL/min/g (p = 0,03) et est resté comparable dans le territoire de la CX (p = 0,3).

**Conclusions:**

le fluage myocardique est un phénomène fréquent lors de l’exécution du stress phramacologique au régadénoson à l’aide du RB-82. Il a un impact significatif sur les valeurs de DSM, en particulier dans le territoire de la CD. La correction du fluage myocardique semble donc nécessaire pour une quantification fiable des flux coronariens.

## Introduction

The use of myocardial blood flow (MBF) quantification using Rubidium-82 (Rb-82) in myocardial perfusion imaging (MPI) with positron emission tomography (PET) is increasing rapidly.^1^-^3^ MPI using Rb-82 PET is of added value in the diagnosis of coronary artery disease, and the MBF quantification provides valuable additional prognostic information about the extent and functional importance of possible stenosis.^4^-^6^

A dynamic PET acquisition including the capture of the first-pass bolus of the activity is required for MBF quantification. Pharmacological vasodilators are generally used to induce stress, while the patient is lying inside the PET scanner.^1^,^7^ The three commonly used vasodilators are adenosine, dipyridamole, and regadenoson. Due to the stimulation of A_1_, A_2B_, and A_3_ receptors, adenosine and dipyridamole are associated with undesirable short-term side-effects as general discomfort, chest pain, and hypotension, and more severe side-effects such as atrioventricular block or bronchospasm.^8^,^9^ An alternative is regadenoson which is a more selective vasodilator that only stimulates A_2A_ receptors and is fast and better tolerated by patients.^10^-^15^ Regadenoson has shown to result in accurate calculation of quantitative MBF values in MPI using Rb-82 PET with similar accuracy compared to adenosine or dipyridamole.^10^,^12^,^16^-^18^ An additional advantage of regadenoson is the significantly lower degree of patient motion compared to adenosine, which can significantly affect the MBF quantification.^19^-^23^

Despite the reduced patient motion when using regadenoson, in clinical practice, we frequently observe repositioning of the heart after administration of regadenoson. This so-called myocardial creep is presumably caused by an increasing respiration and lung volume and thereby the repositioning of the diaphragm and heart after induction of pharmacological stress.^24^ This motion may result in biased MBF measurements and may hamper diagnostic accuracy. Our aim was to determine the percentage of patients with this myocardial creep and to determine its effect on MBF values before and after correction in patients undergoing Rb-82 PET.

## Methods

### Study Design

We retrospectively included 119 consecutive patients referred for MPI using Rb-82 PET/CT (GE Discovery 690, GE Healthcare), who underwent dynamic rest- and pharmacological-induced stress using regadenoson. This study was retrospective and approval by the medical ethics committee was therefore not required according to Dutch law. Nevertheless, all patients provided written informed consent for the use of data for research purposes.

### Patient Preparation and Data Acquisition

All subjects were asked to abstain from caffeine-containing substances for 24 hours and to discontinue dipyridamole-containing medication for 48 hours before imaging. Prior to MPI, a low-dose CT scan was acquired during free-breathing to provide an attenuation map of the chest. This scan was made using a 5-mm slice thickness, 0.8 s rotation time, pitch of 0.97, collimation of 32x0.625 mm, tube voltage of 120 kV, and a tube current of 10 mA. Next, 740 MBq Rb-82 was administered intravenously with a flow rate of 50 mL/min using a Sr-82/Rb-82 generator (CardioGen-82, Bracco Diagnostics Inc.). After the first elution, we induced pharmacological stress by administrating 400 µg (5 mL) of regadenoson over 10 seconds. After a 5 mL saline flush (NaCl 0.9%), we administered a second dose of 740 MBq Rb-82. We acquired seven-minute PET list-mode acquisitions after both Rb-82 administrations. Attenuation correction was applied to all data on the PET system after semiautomatic registration of CT and PET data. We reconstructed the dynamic datasets using 26 time frames (12 × 5 s, 6 × 10 s, 4 × 20 s and 4 × 40 s) with default settings as recommended by the manufacturer using 3D iterative reconstruction using 2 iterations and 24 subsets, while correcting for decay, attenuation, scatter and random coincidences, and dead time effects. Neither time-of-flight information, nor a post-processing filter or resolution modeling was used. Static images were reconstructed from 2:30 to 7:00 minutes for both rest and stress scans.

### Data Processing

The reconstructed dynamic images were processed using Corridor4DM software (v2015.02.64). Myocardium contours were automatically detected in both rest and stress scans based on the static images. Furthermore, a region of interest (ROI) was manually placed at the location of the mitral valve to estimate the activity in the blood pool. The activity concentrations in the myocardium contour and ROI were measured in the 26 reconstructed time frames to calculate the time activity curves (TACs) for the left ventricle (LV), for the three vascular territories: left anterior descending (LAD), left circumflex (LCX) and right coronary (RCA) artery, and for the whole myocardium. The one-tissue compartment model of Lortie et al. based on a ROI methodology was used to calculate the MBF from the TACs using Corridor4DM.^25^

The activity in the myocardium was visually compared with the drawn contours in all individual time frames to detect possible patient motion or myocardial creep. Myocardial creep was defined as gradual decreasing misalignment of the drawn myocardium contour with the activity present in the ventricle and/or myocardium, primarily in the inferior direction. This misalignment was at least one third of the width of the left ventricular myocardial wall and present in at least two time frames of which one had to include the first-pass phase: the filling of the LV. If myocardial creep was present, manual realignment of the contour to the activity in the myocardium was applied in each of the related time frames. Motion not fulfilling the requirements of myocardial creep, suggesting general patient motion, was manually corrected by realigning the myocardium contour to the activity. Patients were excluded when patient motion was present together with myocardial creep to prevent biased results due to overlapping motion. Furthermore, patients with an unreliable TAC were also excluded. Unreliable TACs were defined as TACs showing no clear LV peak.^26^

To evaluate the influence of myocardial creep correction, both rest and stress MBFs were calculated for the original data and for the corrected data regarding the three vascular territories (LAD, LCX, and RCA) and for the whole myocardium. Furthermore, the myocardial flow reserve (MFR), defined as the stress MBF divided by the rest MBF was calculated as well. A difference in MBF or MFR >10% between the corrected and uncorrected scans was considered to possibly influence diagnostic interpretation.

### Statistical Analysis

Patient-specific parameters and characteristics were determined as percentage or mean ± standard deviation (SD) and compared with Chi-square and t-tests as appropriate, using SPSS Statistics version 22.0 (IBM Corporation). The MBF and MFR of the uncorrected and corrected data were compared using the Wilcoxon signed rank test. The level of statistical significance was set to 0.05 for all statistical analyses.

## Results

**Figure 1:**
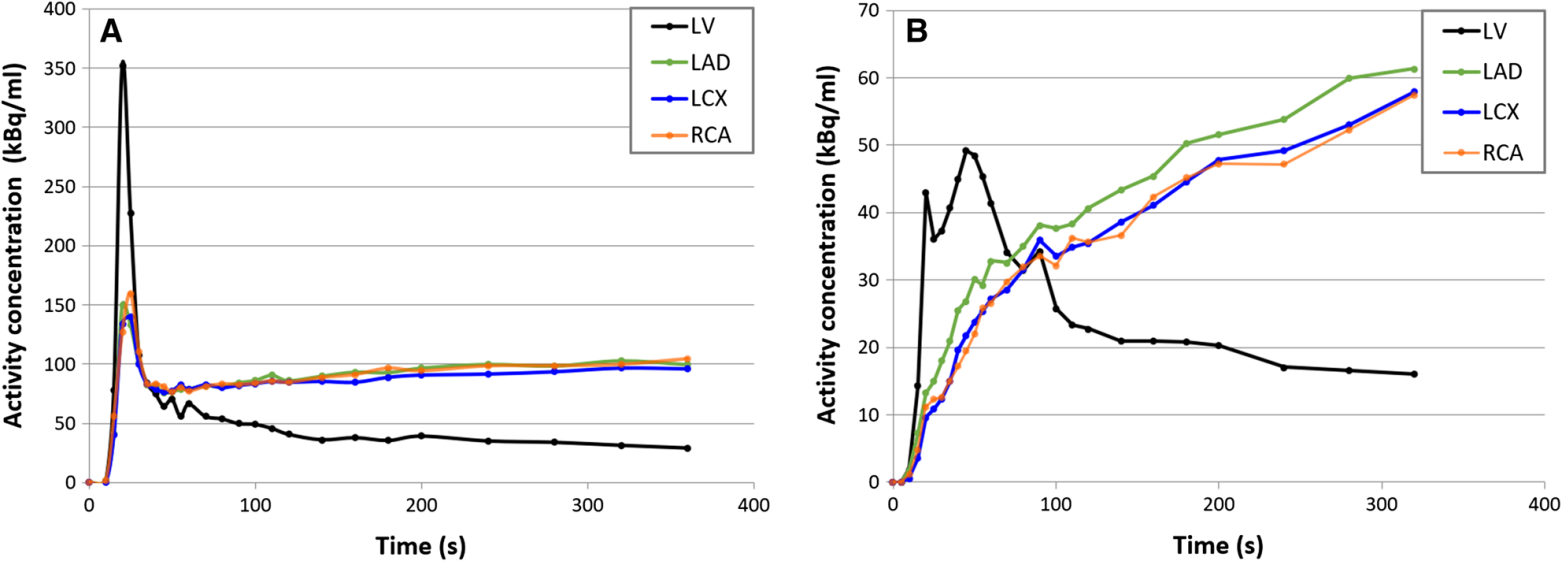
Linegraph showing (**A**) normal time activity curves (TACs) with a high peak value for the left ventricle (LV) during the first-pass phase and where the vascular territories (LAD, LCX and RCA) gradually reach a steady state and (**B**) unreliable TACs with no clear LV peak and lack of steady state for the three vascular territories

Of the 119 patients, 11 (9%) were excluded due to the presence of both patient motion and myocardial creep in the stress data. An additional four patients (3%) were excluded due to unreliable TACs. An example of an unreliable TAC is shown in Figure 1. Of the remaining 104 patients, four (3%) showed only general patient motion in stress.

**Figure 2:**
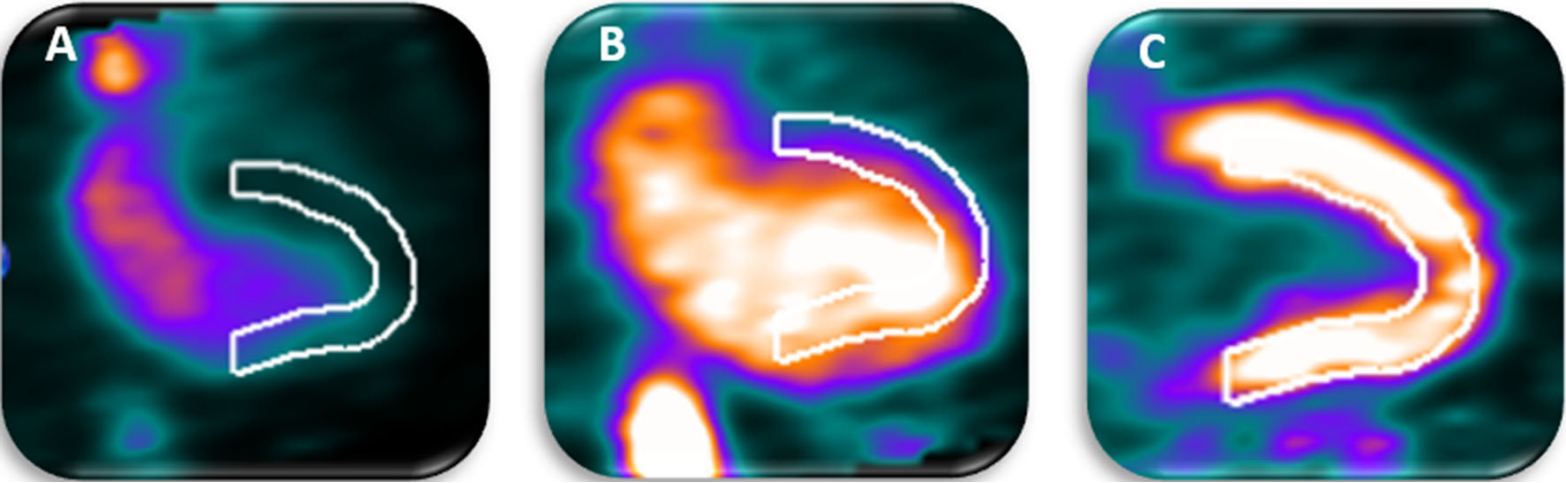
Example of a dynamic Rb-82 PET scan showing myocardial creep. In **A** (15-19 s after injection), the activity reaches the left ventricle (LV) and a misalignment of the automatically drawn myocardium contour and the activity is observed. In **B** (25-29 s after injection), the activity has reached the LV and the myocardium but the misalignment of the drawn myocardium contour and the activity is still observed. In **C** (360-420 s after injection), activity is only present in the myocardium and the heart has returned to its original position resulting in alignment of the observed activity and myocardium contour

The baseline characteristics of the remaining 104 patients are summarized in Table 1. 54 (52%) Patients showed a myocardial creep during the stress scan, as illustrated in Figure 2. Patients with and without myocardial creep did not differ regarding gender, weight, body mass index (BMI), cardiac risk factors and scan outcomes (*P *≥ 0.10). Yet patients with myocardial creep were younger (64 years old) than patients without myocardial creep (70 years old, *P *= 0.004). Of the 54 patients with myocardial creep during stress, two patients also showed myocardial creep during the rest scan.

**Table 1:** Baseline characteristics and scan outcomes of all included patients (*n *= 104) who underwent clinically indicated Rb-82 PET MPI

	Patients with myocardial creep (*n *= 54)	Patients without myocardial creep (*n *= 50)	*P* values (*t* test/*χ*^2^)
Age (years)	64 ± 11	70 ± 11	0.004
Male gender (%)	67	64	0.78
Weight (kg)	90 ± 15	85 ± 18	0.17
Length (cm)	175 ± 9	173 ± 10	0.32
BMI (kg/m^2^)	29.3 ± 4.1	28.5 ± 5.8	0.44
Current smoker (%)	30	16	0.10
Hypertension (%)	46	50	0.71
Diabetes (%)	17	20	0.66
Dyslipidemia (%)	56	50	0.57
Family history (%)	69	54	0.13
Normal MPI scan (%)	76	64	0.18
Ischemic defects on MPI (%)	17	28	0.29
Non-reversible defects on MPI (%)	9	16	0.61

Data are presented as mean ± SD or as percentage

**Figure 3:**
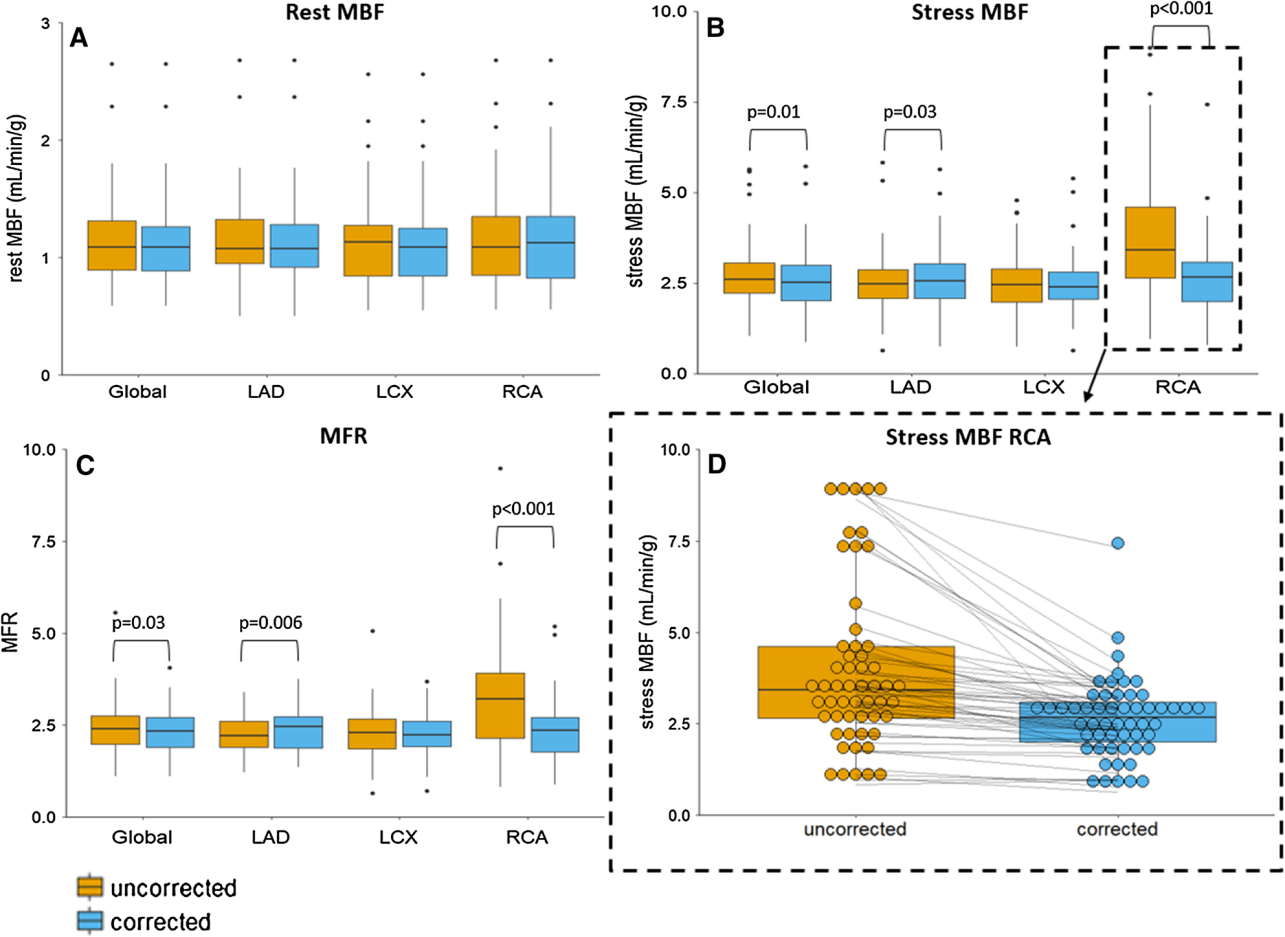
Boxplots showing (**A**) the rest and (**B**) stress myocardial blood flows (MBFs) and (**C**) myocardial flow reserves (MFRs) for the three vascular territories and for the whole myocardium (Global) for the 54 uncorrected and myocardial creep corrected-scans. (**D**) The stress MBF of the RCA with each point representing one patient scan before and after correction showing MBF decreases in 91% (49/54) of the patients after correction

The uncorrected and corrected MBF and MFR measurements, in both rest and stress, for each of the three territorial segments and for the myocardium as a whole (global result) are shown in Table 2 and Figure 3. When comparing the uncorrected and corrected data, the largest differences were found for the RCA territory where the mean MBF decreased from 4.0 to 2.7 mL/min/g (*P* < 0.001) and the mean MFR from 3.5 to 2.4 (*P *< 0.001). Moreover, the MBF of the RCA decreased in 91% (49/54) of the patients, and the MFR of the RCA decreased in 89% (48/54) of the patients, as shown in Figure 3D. Furthermore, differences in MBF and MFR were found for the LAD territory and for the whole myocardium. The mean MBF increased for the LAD from 2.5 to 2.6 mL/min/g (*P *= 0.03) and for the MFR from 2.2 to 2.3 (*P *= 0.006), and for the whole myocardium, the mean MBF and MFR values decreased from 2.7 to 2.6 mL/min/g (*P *= 0.01) and from 2.4 to 2.3 (*P *= 0.03), respectively. No significant differences were found for the LCX territory in stress (*P *= 0.3) nor in the rest scans (*P *≥ 0.11). In the 54 patients with myocardial creep, 45 (83%) had a change > 10% in MBF and 45 (83%) had a change > 10% in MFR in one of the territories or the whole myocardium.

**Table 2:** Uncorrected and corrected rest and stress MBF (mL/min/g) and MFR values for the three vascular territories (LAD, LCX, and RCA) and the whole myocardium (Global)

Vessel		Rest MBF	Stress MBF	MFR
LAD	Uncorrected	1.2 ± 0.4 (0.5 to 2.7)	2.5 ± 0.9 (0.7 to 5.8)	2.2 ± 0.5 (1.2 to 3.4)
	Corrected	1.2 ± 0.4 (0.5 to 2.7)	2.6 ± 0.9* (0.8 to 5.6)	2.3 ± 0.6** (1.4 to 3.8)
LCX	Uncorrected	1.1 ± 0.4 (0.6 to 2.6)	2.5 ± 0.9 (0.8 to 4.8)	2.3 ± 0.7 (0.7 to 5.1)
	Corrected	1.1 ± 0.4 (0.6 to 2.6)	2.5 ± 0.8 (0.7 to 5.4)	2.3 ± 0.6 (0.7 to 3.7)
RCA	Uncorrected	1.2 ± 0.5 (0.6 to 2.7)	4.0 ± 2.3 (1.0 to 9.0)	3.5 ± 1.9 (0.8 to 11)
	Corrected	1.2 ± 0.4 (0.6 to 2.7)	2.7 ± 1.1*** (0.8 to 7.4)	2.4 ± 0.8*** (0.9 to 5.2)
Global	Uncorrected	1.2 ± 0.4 (0.6 to 2.7)	2.7 ± 1.0 (1.0 to 5.7)	2.4 ± 0.7 (1.1 to 5.6)
	Corrected	1.1 ± 0.4 (0.6 to 2.7)	2.6 ± 0.9* (0.9 to 5.7)	2.3 ± 0.6* (1.1 to 4.1)

Data are presented as mean ± SD

*LAD*, left anterior descending; *LCX*, left circumflex; *MBF*, myocardial blood flow; *MFR*, myocardial flow reserve; *RCA*, right coronary artery

*P < 0.05; *P < 0.01; ***P < 0.001

## Discussion

**Figure 4:**
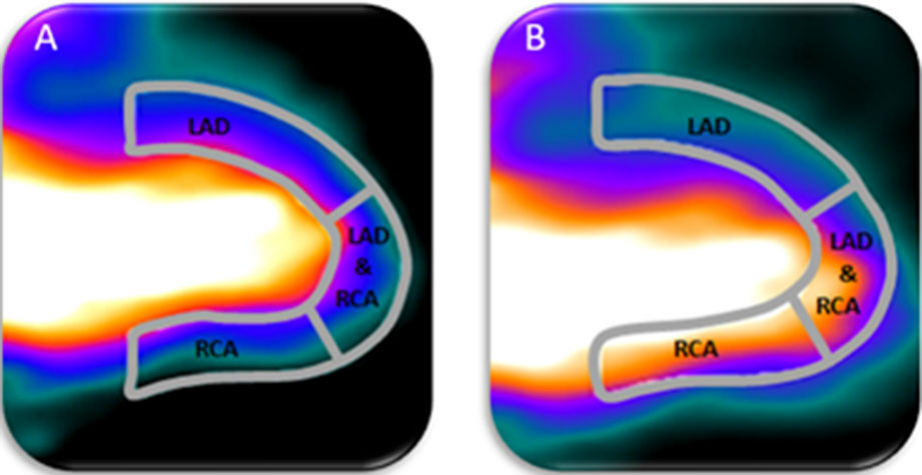
Proper alignment of the automatically drawn myocardium contour and the activity in the heart is shown in **A**. In case of myocardial creep, there is a misalignment of the drawn myocardium contour with the activity in the heart, as shown in **B**. This results in increased measured activity in the RCA and partly in the LAD territory

In this study, we have demonstrated that a myocardial creep occurs in more than half of the patients during regadenoson-induced stress MPI using Rb-82 PET. Moreover, correction of this myocardial creep resulted in significantly lower MBF and MFR values for the RCA territory and may improve diagnostic accuracy. Besides the large impact on MBF and MFR values in the RCA territory, myocardial creep also resulted in significant differences in stress MBF and MFR values for the LAD and the whole myocardium. These differences can be explained by the anatomic position and direction of myocardial creep, as illustrated in Figure 4. During the first-pass phase when the Rb-82 activity is in the LV, there is a strong overlap between the activity and the part of the myocardium contour that is perfused by the RCA and to a lesser extent by the LAD when myocardial creep is present. After correction, the overlap diminishes, which directly affects the MBF and MFR measurements.

Multiple studies have reported the occurrence of myocardial creep, also known as non-returning motion of the heart, primarily occurring in the post-stress period during MPI using different pharmacological vasodilators.^19^,^23^,^24^,^27^,^28^ A recent study by Memmot et al. reported a non-returning motion or myocardial creep in 36% (11/30) of their patients during MPI using Rb-82 PET and regadenoson as vasodilator independent of age.^19^ This percentage is in fair agreement with the 52% found in this study, although we used a different methodology to assess the presence of myocardial creep and a slightly different time-framing combination. Furthermore, they showed that 69% (11/16) of the patients stressed with regadenoson with visible motion were categorized as myocardial creep which is in fair agreement to the 78% (54/69) found in our study. Moreover, they reported that only 10% (3/30) of their patients showed significant motion, which was defined as motion greater than half the width of the myocardial wall. Although we did not assess severity or amount of myocardial creep, we did observe that correcting for myocardial creep majorly affected the MBF quantification in most patients and presumably also in patients with only a limited amount of myocardial creep. Lee et al. recently reported that greater motion was observed during stress, especially in the inferior direction which reflects myocardial creep which is in high agreement with our study.^28^ They also reported that motion resulted in the largest changes in the MBF and MFR in the RCA territory, consistent with our results.

Multiple mechanisms are hypothesized in the literature to explain the occurrence of myocardial creep. Karacalioglu et al. hypothesised that myocardial creep is caused by gravity on the organs when patients switch from a standing to a lying position in the scanner. They reported that a five-minute bed rest on the scanner table significantly decreased the vertical motion of the heart.^29^ A CT-scan followed by the rest scan was performed before the stress scan in our protocol. Therefore, the mechanism described above does not explain the myocardial creep we found during stress imaging. Although this gravity theory might explain myocardial creep during rest acquisitions, we observed myocardial creep in only 2% of the rest scans and therefore think this is most likely caused by anxiety at the start of a MPI scan.^30^

Another mechanism previously described by Friedman et al. which is more likely to cause myocardial creep is that after administration of a pharmacological vasodilator, in our case regadenoson, lung volume increases which causes a repositioning of the diaphragm and heart.^24^ Hence, we are unable to prevent this repositioning of the heart and thus the occurrence of myocardial creep.

Several limitations of this study should be recognized. First, we were unable to determine the effect of myocardial creep correction on the diagnostic accuracy due to the lack of a reference standard. However, in some patients myocardial creep resulted in unrealistic high MBF values (>5 mL/min/g) which decreased after correction to realistic values. Hence, we assume that correcting for myocardial creep increases diagnostic reliability.

Second, manual actions are required in the quantification process and for the myocardial creep correction which could have introduced additional operator variability. Although this operator variability might have introduced additional variance, the changes in stress MBF quantification were higher than the previously reported ± 10% test-re-test reproducibility errors when calculating the MBF using Rb-82 PET in MPI.^31^ Thus, the operator variability is expected to be of limited influence.

Third, a high fraction of the patients had a normal MBF, possibly limiting generalization. However, in case of the poorly perfused tissue with myocardial creep, the influence of spillover from the LV is expected to be larger than that for the normal perfused tissue resulting in a relatively larger overestimation of the modeling parameter k1 and, hence, MBF.^28^ This could result in larger differences between MBF values in the RCA territory before and after myocardial creep correction than those reported in this study.

Finally, we only corrected the myocardial creep in the attenuation-corrected PET images. However, only the PET data acquired between 2:30 and 7:00 minutes were co-registered to the CT to create an attenuation map. As myocardial creep only occurs in the earlier time frames, misregistration and, hence, attenuation-correction artifacts may occur. This misregistration could result in altered MBF measurements.^32^-^35^ Adding a second low-dose CT-scan immediately before the stress PET acquisition is unlikely to improve PET/CT registration as the myocardial creep misregistration occurs after induction of stress and is only temporary. However, we believe that frame-based co-registration of the stress-PET and CT data can improve PET/CT registration and thereby the reliability of Rb-82 PET quantification in patients with myocardial creep.^28^

## New Knowledge Gained

If myocardial creep is present but remains uncorrected in clinical practice, the stress MBF and MFR of the RCA territory will be overestimated, as shown in Figure 3D, which can lead to incorrect diagnosis. The MFR of the RCA may fall within the normal range of the MFR values (> 1.7), while after correcting for myocardial creep, the MFR drops below this threshold, affecting the diagnosis.^3^ Moreover, Memmot et al. showed that myocardial creep occurs more frequently when adenosine is used as pharmacological vasodilator (96%) in comparison with regadenoson (69%).^19^ Therefore, we strongly recommend to check the presence of myocardial creep in all patients regardless of the used pharmacological vasodilator and correct for it to achieve reliable MBF and MFR measurements.

There are two practical ways to recognize myocardial creep in clinical practice. The first sign is an elevated time activity concentration of the RCA during the first-pass phase in the TAC in comparison with the LCX and LAD. As no activity is yet present in the myocardium, the whole activity measured in this phase is due to spillover and should therefore be constant across the three vascular territories, as shown in Figure 1. The second sign is the misalignment between the automatically drawn myocardium contour and the observed activity during the first-pass phase. As in 83% of our patients with myocardial creep an MBF change >10% occurred after correction, this implies that even a small myocardial creep should be corrected in clinical practice.

## Conclusions

Myocardial creep was seen in 52% of the patients who underwent regadenoson-induced stress Rb-82 PET. Correcting for myocardial creep significantly changed MBF measurements during stress and MFR quantification, especially in the RCA territory. As this may hamper diagnostic accuracy, detection and correction of myocardial creep seem necessary for reliable quantification when using regadenoson.

## Electronic supplementary material

Below is the link to the electronic supplementary material.

Supplementary material 1 (PPTX 3170 kb)

## Abbreviations

BMI: Body mass index
LAD: Left anterior descending
LCX: Left circumflex
MBF: Myocardial blood flow
MFR: Myocardial flow reserve
MPI: Myocardial perfusion imaging
PET: Positron emission tomography
Rb-82: Rubidium-82
RCA: Right coronary artery
TAC: Time activity curve
