# Supplementary material for: Impact of regadenoson-induced myocardial creep on dynamic Rubidium-82 PET myocardial blood flow quantification
Source: J Nucl Cardiol. 2019 Feb 20;26(3):719–28. doi: 10.1007/s12350-019-01649-4 (PMC6517358; doi:10.1007/s12350-019-01649-4)
Supplement: Supplementary file 1 — Supplementary material 1 (PPTX 3170 kb) [file 12350_2019_1649_MOESM1_ESM.pptx]

## Slide 1
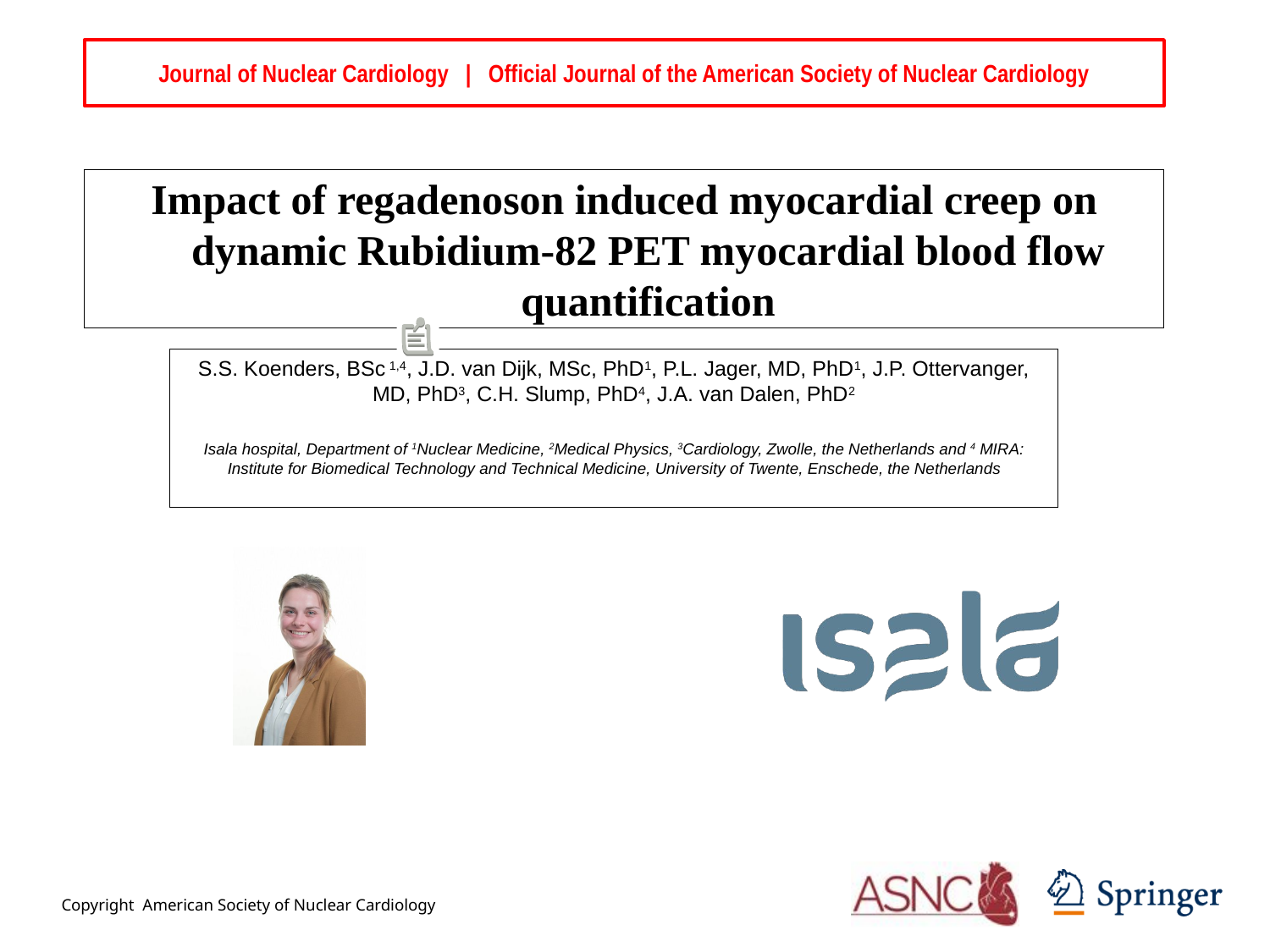

Journal of Nuclear Cardiology | Official Journal of the American Society of Nuclear Cardiology
# Impact of regadenoson induced myocardial creep on dynamic Rubidium-82 PET myocardial blood flow quantification
S.S. Koenders, BSc 1,4, J.D. van Dijk, MSc, PhD1, P.L. Jager, MD, PhD1, J.P. Ottervanger, MD, PhD3, C.H. Slump, PhD4, J.A. van Dalen, PhD2
Isala hospital, Department of 1Nuclear Medicine, 2Medical Physics, 3Cardiology, Zwolle, the Netherlands and 4 MIRA: Institute for Biomedical Technology and Technical Medicine, University of Twente, Enschede, the Netherlands
Copyright American Society of Nuclear Cardiology

## Slide 2
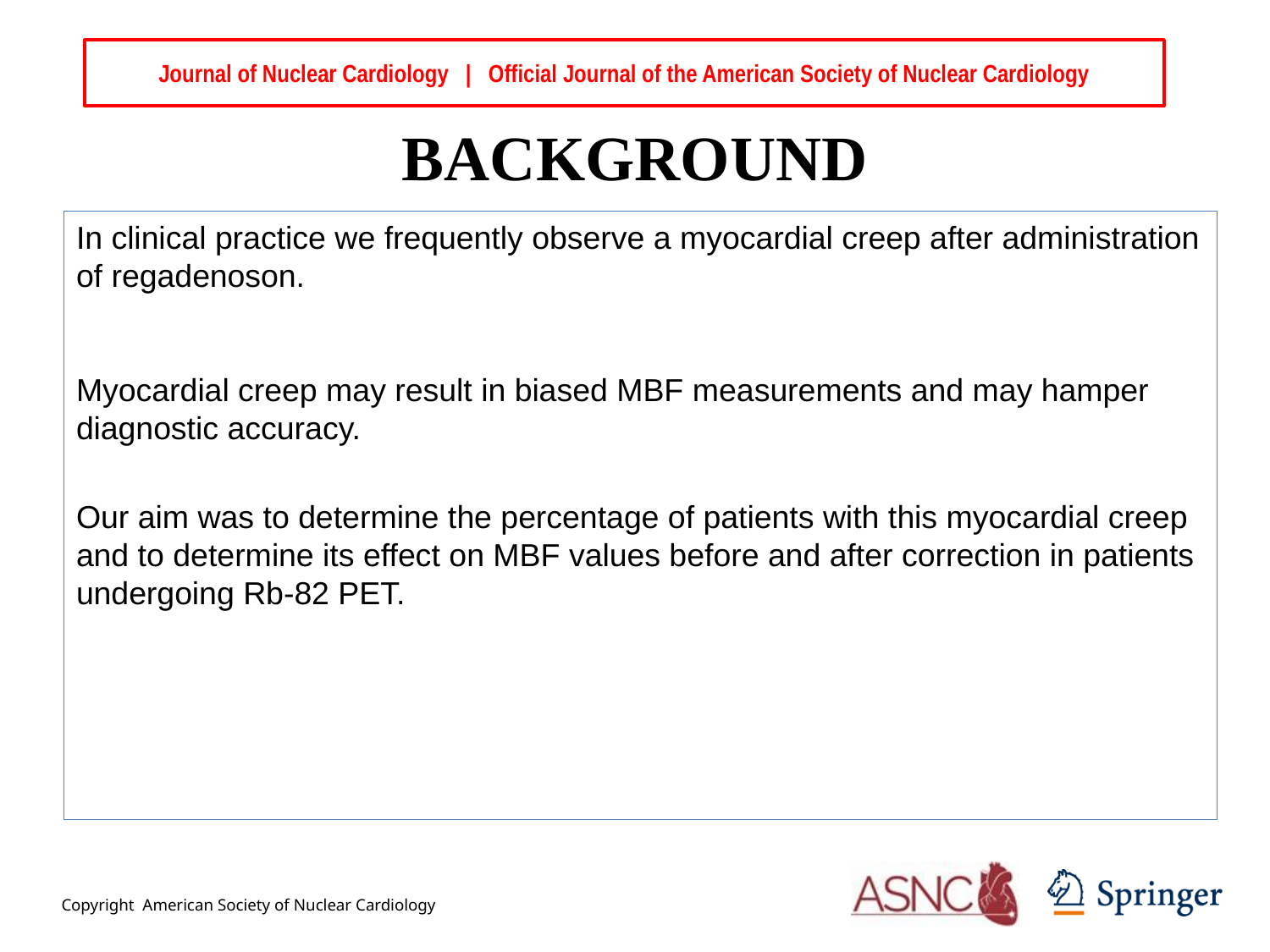

Journal of Nuclear Cardiology | Official Journal of the American Society of Nuclear Cardiology
# BACKGROUND
In clinical practice we frequently observe a myocardial creep after administration of regadenoson.
Myocardial creep may result in biased MBF measurements and may hamper diagnostic accuracy.
Our aim was to determine the percentage of patients with this myocardial creep and to determine its effect on MBF values before and after correction in patients undergoing Rb-82 PET.
Copyright American Society of Nuclear Cardiology

## Slide 3
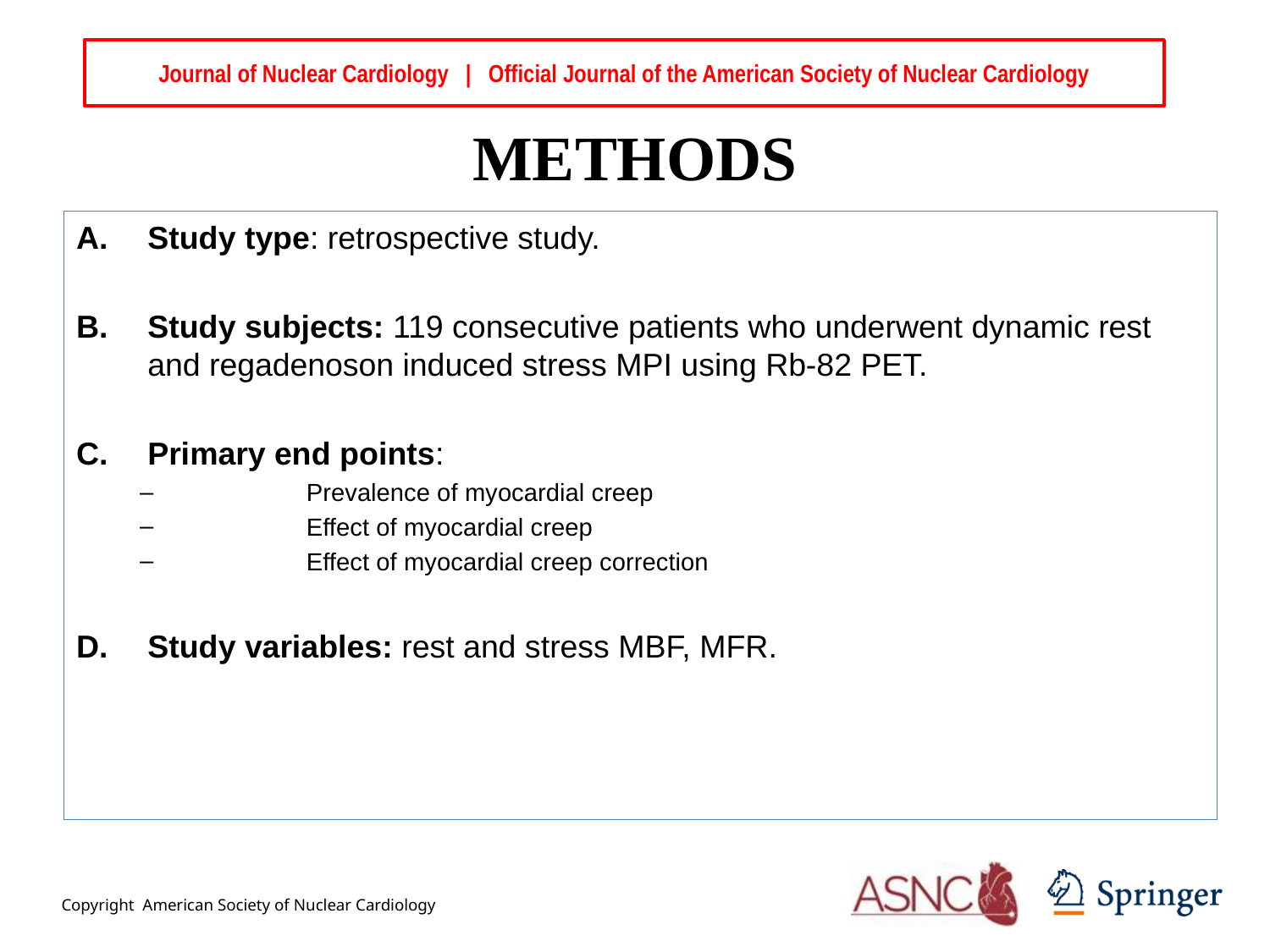

Journal of Nuclear Cardiology | Official Journal of the American Society of Nuclear Cardiology
# METHODS
Study type: retrospective study.
Study subjects: 119 consecutive patients who underwent dynamic rest and regadenoson induced stress MPI using Rb-82 PET.
Primary end points:
	Prevalence of myocardial creep
	Effect of myocardial creep
	Effect of myocardial creep correction
Study variables: rest and stress MBF, MFR.
Copyright American Society of Nuclear Cardiology

## Slide 4
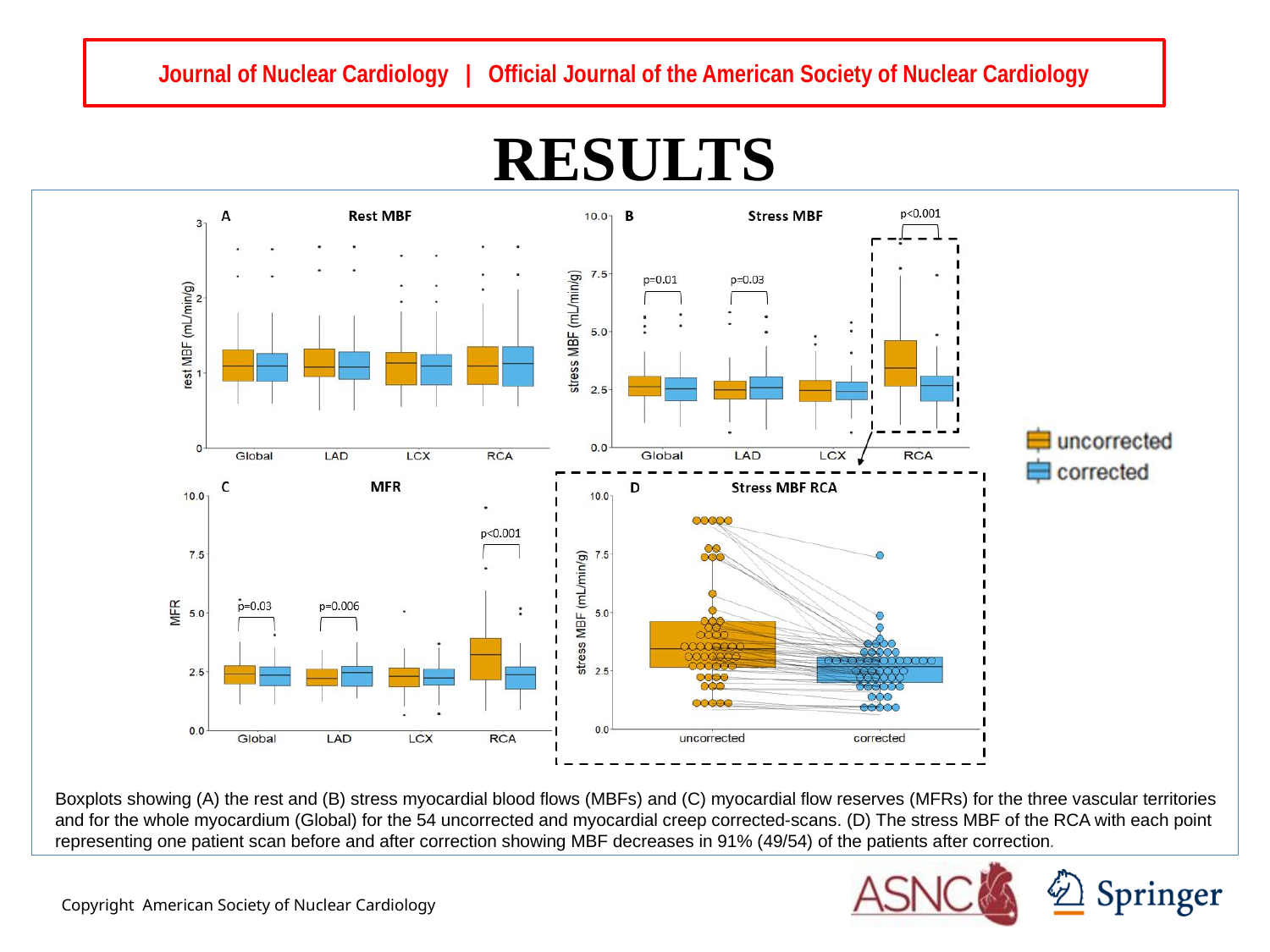

Journal of Nuclear Cardiology | Official Journal of the American Society of Nuclear Cardiology
# RESULTS
Boxplots showing (A) the rest and (B) stress myocardial blood flows (MBFs) and (C) myocardial flow reserves (MFRs) for the three vascular territories and for the whole myocardium (Global) for the 54 uncorrected and myocardial creep corrected-scans. (D) The stress MBF of the RCA with each point representing one patient scan before and after correction showing MBF decreases in 91% (49/54) of the patients after correction.
Copyright American Society of Nuclear Cardiology

## Slide 5
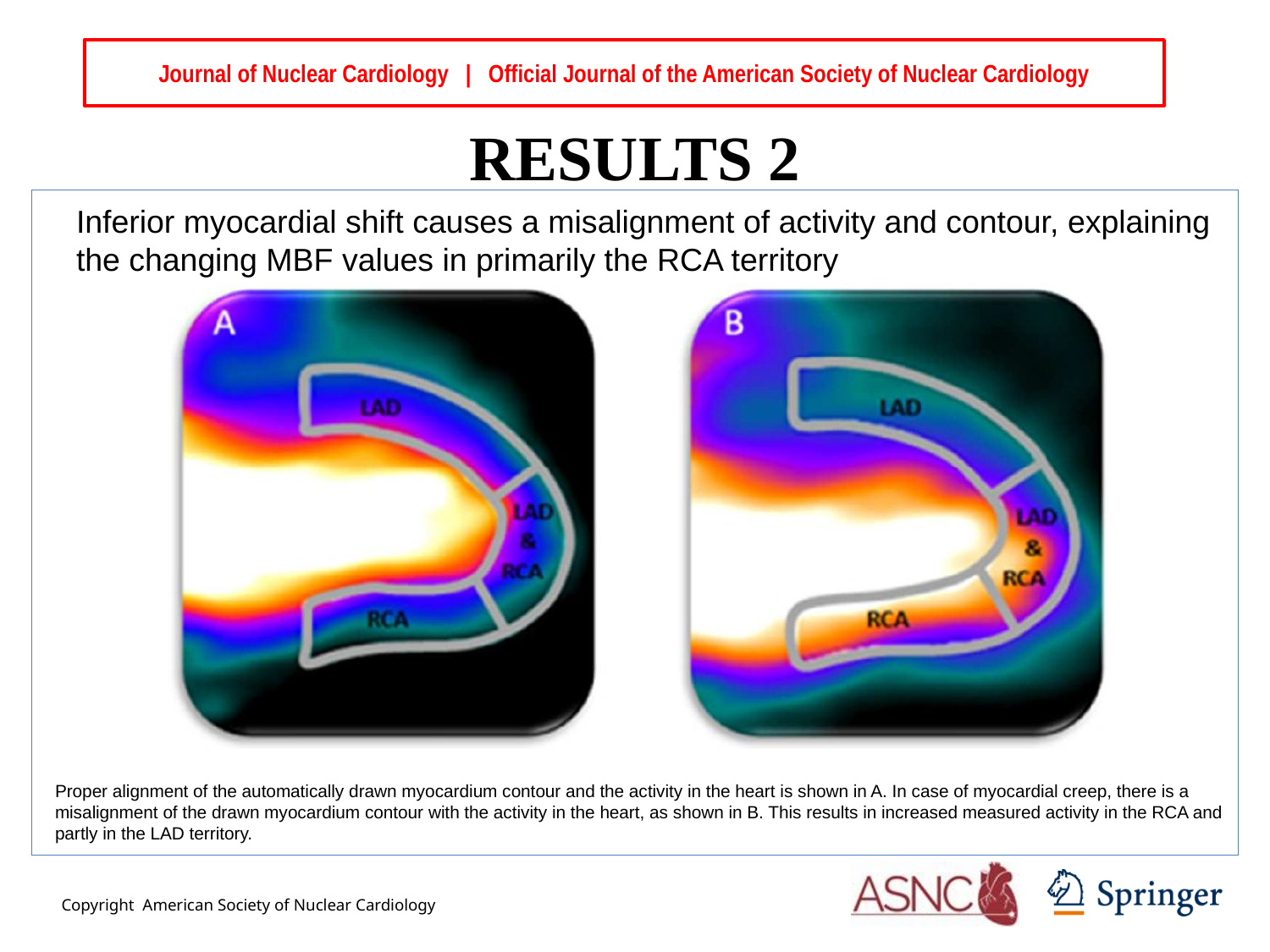

Journal of Nuclear Cardiology | Official Journal of the American Society of Nuclear Cardiology
# RESULTS 2
Inferior myocardial shift causes a misalignment of activity and contour, explaining the changing MBF values in primarily the RCA territory
Proper alignment of the automatically drawn myocardium contour and the activity in the heart is shown in A. In case of myocardial creep, there is a misalignment of the drawn myocardium contour with the activity in the heart, as shown in B. This results in increased measured activity in the RCA and partly in the LAD territory.
Copyright American Society of Nuclear Cardiology

## Slide 6
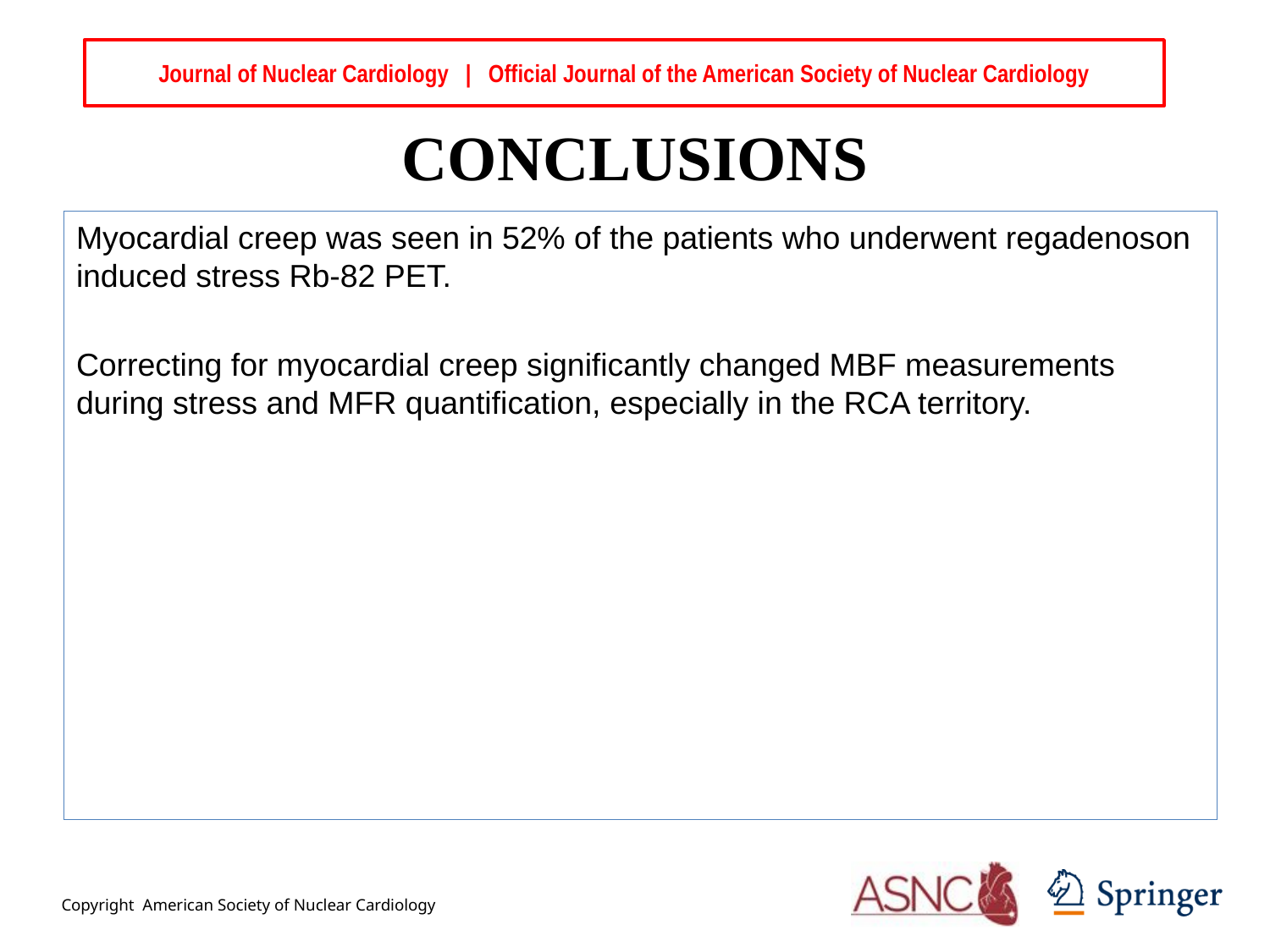

Journal of Nuclear Cardiology | Official Journal of the American Society of Nuclear Cardiology
# CONCLUSIONS
Myocardial creep was seen in 52% of the patients who underwent regadenoson induced stress Rb-82 PET.
Correcting for myocardial creep significantly changed MBF measurements during stress and MFR quantification, especially in the RCA territory.
Copyright American Society of Nuclear Cardiology
